# Convalescent Plasma for Hospitalized COVID-19 Patients: A Single-Center Experience

**DOI:** 10.3390/life12030420

**Published:** 2022-03-14

**Authors:** Massimo Franchini, Claudia Glingani, Giuseppe De Donno, Giuseppe Lucchini, Massimiliano Beccaria, Massimo Amato, Gian Paolo Castelli, Leonardo Bianciardi, Mauro Pagani, Marco Ghirardini, Giuseppe Puma, Barbara Presciuttini, Maria Teresa Costantino, Marilena Frigato, Verena Crosato, Giorgio Tiecco, Alice Mulè, Dorothea Angela Papalia, Francesco Inglese, Fabio Spreafico, Martina Garuti, Antonietta Pecoriello, Giulia Cervi, Graziana Greco, Vanni Galavotti, Tiziana Santini, Angela Berselli, Carlo Montalto, Riccardo Bertoletti, Simona Aurelia Bellometti, Enrico Capuzzo, Dario Benazzi, Gianpaolo Grisolia, Fabio Pajola, Raffaello Stradoni, Matteo Zani, Adriano Verzola, Vito Codeluppi, Silvia Vesentini, Elisa Bellocchio, Marco Candini, Giorgina Ambrosi, Francesca Carandina, Cleante Scarduelli, Albino Reggiani, Salvatore Casari, on behalf of Convalescent Plasma Study Group

**Affiliations:** 1 Department of Transfusion Medicine and Hematology, Carlo Poma Hospital, Azienda Socio Sanitaria Territoriale of Mantova, 46100 Mantova, Italy; claudia.glingani@asst-mantova.it (C.G.); enrico.capuzzo@asst-mantova.it (E.C.); matteo.zani@asst-mantova.it (M.Z.); 2 Intensive Care Respiratory Unit, Carlo Poma Hospital, Azienda Socio Sanitaria Territoriale of Mantova, 46100 Mantova, Italy; giuseppe.dedonno@asst-mantova.it (G.D.D.); massimiliano.beccaria@asst-mantova.it (M.B.); francesco.inglese@asst-mantova.it (F.I.); fabio.spreafico@asst-mantova.it (F.S.); martina.garuti@asst-mantova.it (M.G.); antonietta.pecoriello@asst-mantova.it (A.P.); giulia.cervi@asst-mantova.it (G.C.); graziana.greco@asst-mantova.it (G.G.); vanni.galavotti@asst-mantova.it (V.G.); 3 Biostatistic Unit, Carlo Poma Hospital, Azienda Socio Sanitaria Territoriale of Mantova, 46100 Mantova, Italy; giuseppe.lucchini@asst-mantova.it; 4 Emergency Department, Carlo Poma Hospital, Azienda Socio Sanitaria Territoriale of Mantova, 46100 Mantova, Italy; massimo.amato@asst-mantova.it (M.A.); dario.benazzi@asst-mantova.it (D.B.); 5 Department of Anesthesiology and Intensive Care, Carlo Poma Hospital, Azienda Socio Sanitaria Territoriale of Mantova, 46100 Mantova, Italy; gianpaolo.castelli@asst-mantova.it (G.P.C.); angela.berselli@asst-mantova.it (A.B.); carlo.montalto@asst-mantova.it (C.M.); 6 Anesthesiology and Intensive Care Unit, SS Trinità and San Marcellino Muravera (Cagliari) Hospital, ASL 8 Cagliari, 09043 Cagliari, Italy; leonardo.bianciardi@atssardegna.it; 7 Internal Medicine Unit, Department of Medicine, Carlo Poma Hospital, Azienda Socio Sanitaria Territoriale of Mantova, 46100 Mantova, Italy; mauro.pagani@asst-mantova.it (M.P.); barbara.presciuttini@asst-mantova.it (B.P.); 8 Department of Medicine, Hospital of Asola, Azienda Socio Sanitaria Territoriale of Mantova, 46100 Mantova, Italy; marco.ghirardini@asst-mantova.it (M.G.); tiziana.santini@asst-mantova.it (T.S.); 9 Unit of Infectious Diseases, Carlo Poma Hospital, Azienda Socio Sanitaria Territoriale of Mantova, 46100 Mantova, Italy; giuseppe.puma@asst-mantova.it (G.P.); salvatore.casari@asst-mantova.it (S.C.); 10 Allergology and Clinical Immunology Unit, Department of Medicine, Carlo Poma Hospital, Azienda Socio Sanitaria Territoriale of Mantova, 46100 Mantova, Italy; mariateresa.costantino@asst-mantova.it (M.T.C.); marilena.frigato@asst-mantova.it (M.F.); 11 Infectious and Tropical Diseases Clinic, University of Brescia and Azienda Socio Sanitaria Territoriale Spedali Civili, 25123 Brescia, Italy; v.crosato@unibs.it (V.C.); g.tiecco@unibs.it (G.T.); a.mule@unibs.it (A.M.); d.papalia@unibs.it (D.A.P.); 12 Medical Direction, Azienda Socio Sanitaria Territoriale of Mantova, 46100 Mantova, Italy; riccardo.bertoletti@asst-mantova.it (R.B.); simona.bellometti@asst-mantova.it (S.A.B.); fabio.pajola@asst-mantova.it (F.P.); 13 Department of Obstetrics and Gynecology, Carlo Poma Hospital, Azienda Socio Sanitaria Territoriale of Mantova, 46100 Mantova, Italy; gianpaolo.grisolia@asst-mantova.it; 14 General Direction, Azienda Socio Sanitaria Territoriale of Valcamonica, 25043 Breno, Italy; raffaello.stradoni@ats-montagna.it; 15 Management Planning and Control Service, Carlo Poma Hospital, Azienda Socio Sanitaria Territoriale of Mantova, 46100 Mantova, Italy; adriano.verzola@asst-mantova.it; 16 Department of Anesthesiology and Intensive Care, Destra Secchia Hospital, Azienda Socio Sanitaria Territoriale of Mantova, 46100 Mantova, Italy; vito.codeluppi@asst-mantova.it (V.C.); silvia.vesentini@asst-mantova.it (S.V.); elisa.bellocchio@asst-mantova.it (E.B.); marco.candini@asst-mantova.it (M.C.); giorgina.ambrosi@asst-mantova.it (G.A.); francesca.carandina@asst-mantova.it (F.C.); 17 Intensive Cardiopulmonary Rehabilitation Unit, Carlo Poma Hospital, Azienda Socio Sanitaria Territoriale of Mantova, 46100 Mantova, Italy; cleante.scarduelli@asst-mantova.it; 18 Cardiology Unit, Destra Secchia Hospital, Azienda Socio Sanitaria Territoriale of Mantova, 46100 Mantova, Italy; albino.reggiani@asst-mantova.it

**Keywords:** COVID-19, convalescent plasma, mortality, safety

## Abstract

In Winter 2020, Italy, and in particular the Lombardy region, was the first country in the Western hemisphere to be hit by the COVID-19 pandemic. Plasma from individuals recovered from COVID-19 (COVID-19 convalescent plasma, CCP) was the first therapeutic tool adopted to counteract the severe acute respiratory syndrome coronavirus-2 (SARS-CoV-2). In this retrospective cohort study, we report the experience of the city hospital of Mantua, Lombardy region, on the compassionate use of CCP in patients hospitalized for severe COVID-19. Between April 2020 and April 2021, 405 consecutive COVID-19 patients received 657 CCP units with a median anti-SARS-CoV-2 neutralizing antibody (nAb) titer of 160 (interquartile range (IQR), 80–320). Their median age was 68 years (IQR, 56–78 years), and 62% were males. At enrollment, 55% of patients had an increased body mass index (BMI), and 25.6% had at least three comorbidities. The 28-day crude mortality rate was 12.6% (51/405). Young age (<68 years), mild disease (admission to low-intensity departments) and early treatment (<7 days from symptoms onset) with high nAb titer (≥320) CCP were found as independently associated with a favorable response to CCP treatment. No safety concerns were recorded, with a rate of CCP-related adverse reactions (all of mild intensity) of 1.3%. In our real-life experience, the first in the western world, early administration of high-titer CCP was a safe and effective treatment for hospitalized COVID-19 patients.

## 1. Introduction

Severe acute respiratory syndrome coronavirus 2 (SARS-CoV-2) has caused the coronavirus disease 2019 (COVID-19) pandemic, which was first reported from Wuhan at the end of 2019 and spread all over the world within a few months [1,2]. At the time of writing, more than 6 million people have died from coronavirus disease 2019 (COVID-19), and more than 400 million people have been infected [3]. Clinicians and researchers have struggled to develop an effective therapeutic protocol to treat and contain the spread of this infectious disease, and more than 300 drugs have been or are being investigated under clinical trials in different parts of the world [4,5]. Among the various therapeutic and prophylactic strategies developed to contain the COVID-19 epidemic, passive immunization by transfusion of COVID-19 convalescent plasma (CCP) has been utilized, particularly during the first two pandemic waves, before the introduction into the market of monoclonal antibody (mAb)-based therapies and small-chemical antivirals [6].

The results from randomized and non-randomized controlled trials (RCTs) on CCP have produced mixed results, probably due to the wide heterogeneity in their study designs and patient populations enrolled [7]. Furthermore, these trials were heterogeneous with respect to the characteristics of the CCP used (e.g., in terms of antibody content and stratification of recipients according to their serological status or disease severity). Despite these limitations, there are currently positive signals from the literature on a beneficial effect of CCP when administered early (within 72 h from symptom onset) and with a high-titer (>1:160) of anti-SARS-CoV-2 neutralizing antibodies (nAb) [8,9].

In this study, we reported the results of the one-year experience (April 2020–April 2021) on the compassionate use of CCP in patients hospitalized for severe COVID-19 at the city hospital of Mantua, Italy.

## 2. Patients and Methods

### 2.1. Patient Selection

In this retrospective study, we reported all the consecutive patients admitted to the city hospital of Mantua for COVID-19 and transfused with CCP between 1 April 2020 and 30 April 2021. This period was divided into two phases: the first pandemic wave from April to July 2020 and the second–third pandemic wave from October 2020 to April 2021. The transfusion of CCP was performed within a compassionate use program authorized by both the local ethical committee and the hospital health management. The study was registered at clinicaltrials.gov as NCT05157165.

We enrolled in the CCP compassionate use program adult inpatients with a confirmed diagnosis of SARS-CoV-2 infection (i.e., with a nasopharyngeal swab positive for SARS-CoV-2 by polymerase chain reaction) and at least one of the following criteria indicative of severe COVID-19: (1) radiologically confirmed pneumonia; (2) oxygen saturation (SpO2) ≤ 93% at rest and in-room air; (3) partial pressure of oxygen (PaO2)/fraction of inspired oxygen (FiO2) ≤ 300 mmHg. Written informed consent was obtained before enrollment.

Exclusion criteria were: (1) patients under the age of 18 years; (2) patients with proven hypersensitivity or allergic reaction to plasma, blood product or immunoglobulins.

In addition to patients’ demographic and physical data (age, sex and body mass index (BMI)) and associated drug therapy, patients’ ABO blood group and comorbidities (obesity, arterial hypertension, cardiovascular diseases, chronic kidney disease, dyslipidemia, diabetes mellitus, chronic lung disease and cancer) were also considered. In addition to these parameters, we recorded the date of hospital admission and of discharge (or death), the degree of intensity of the hospital department of admission (low intensity: infectious disease and internal medicine departments, where patients have undergone medical therapy, including oxygen therapy at high flows, but not mechanical ventilation; intermediate-high intensity: respiratory intensive care unit, emergency medicine and intensive care unit where patients have undergone mechanical invasive and non-invasive mechanical ventilation), the date of CCP transfusion, the days between symptom onset and transfusion of the first CCP unit, the anti-SARS-CoV-2 nAb titer and the PaO2/FiO2 ratio before CCP transfusion.

### 2.2. Convalescent Plasma

CCP units were obtained from previously infected subjects who had recovered and cleared the virus. Eligible donors were either men or nulliparous women aged 18 to 65 years, weighing more than 50 kg, with a laboratory-confirmed diagnosis of SARS-CoV-2 infection that had completely resolved at least 14 days before donation, as confirmed by two consecutive negative SARS-CoV-2 PCR test results from nasopharyngeal swabs collected 24 h apart. CCP donors were enrolled on a voluntary basis and had to meet all standard plasma collection requirements as provided by the current Italian laws. All routine screening tests for blood donors, including ABO blood group typing; Rh phenotyping; complete blood count; and screening for human immunodeficiency virus; hepatitis B, C, A and E viruses; parvovirus B19; and syphilis were conducted according to Italian regulations and the indications of the Italian National Blood Center [10].

CCP was collected through a plasmapheresis procedure using the AURORA cell separator (Fresenius Kabi, Italy), processed, and stored in agreement with national CCP regulations [10,11]. A plasma volume of about 600 mL was collected during each procedure and immediately divided into two bags, each corresponding to a therapeutic CCP unit of 300 mL. Collected CCP had an anti-SARS-CoV-2 nAb titer of 1:80 or higher. The live authentic SARS-CoV-2 neutralization test for the titration of nAbs was performed at the Molecular Virology Unit of the University Hospital of Pavia and was based on the determination of cytopathic effect, as previously described [12,13]. Viral inactivation, by using the UVA photoinactivation method following the addition of amotosalen hydrochloride (Intercept-CERUS System, distributed in Italy by Kedrion SpA, Castelvecchio Pascoli, Lucca, Italy) was applied to all CCP units, in accordance with Italian regulation at that time, before freezing 300 mL aliquots.

The CCP transfusions were performed by medical and nursing staff. CCP recipients were transfused with one to three units of ABO type-compatible CCP, according to the clinical response, over a period of 3–5 days. All the procedures were performed in agreement with the routine procedures of the Transfusion Service of the city Hospital of Mantua. All donors provided their written consent after being thoroughly informed.

### 2.3. Outcomes

The primary outcome in our cohort of CCP-treated patients was the overall mortality at 28 days after hospitalization. A subgroup analysis was also performed to identify patient- and treatment-related factors possibly related to a worse prognosis (i.e., sex, age, ABO blood group, BMI, comorbidities, COVID-19 severity (measured as PaO2/FiO2 and intensity of admission department), days between symptom onset CCP transfusion, number and nAb titer of CCP units transfused).

A secondary outcome was the safety of CCP treatment, measured as the rate of adverse reactions to CCP transfusion. The type, degree and outcome of adverse events occurring during or after (within 72 h) CCP transfusion were recorded, according to the Guideline on Good Pharmacovigilance Practices of the European Medicines Agency (https://www.ema.europa.eu/en/documents/scientific-guideline/guideline-good-pharmacovigilance-practices-annex-i-definitions-rev-4_en.pdf, accessed on 19 February 2022). Serious transfusion reactions were defined as a transfusion-associated circulatory overload (TACO), transfusion-related acute lung injury (TRALI), severe allergic reaction, hypotensive reaction or death. By definition, all serious transfusion reactions occurred within 6 h of CCP transfusion. All transfusion reactions were collected using electronic case report forms recorded in the local management software system for transfusion services EmoNet (GPI Group, Trento, Italy). Adverse reactions to CCP transfusion were also recorded in a computer database using the national hemovigilance system of the transfusion network organized by the Italian National Blood Center (SISTRA).

### 2.4. Statistical Analysis

Continuous variables were reported as mean (±standard deviation) or median and interquartile range (IQR) as appropriate according to distribution, while categorical data are reported as numbers and percentages. Comparisons between groups were carried out with an independent *t*-test or Mann–Whitney U test for continuous variables and chi-squared test or Fisher’s exact test for categorical variables, as appropriate. All statistical tests were two-sided, and associations were considered statistically significant when the values were below a nominal level of 0.05 (*p* < 0.05).

The multivariate analysis was conducted with the binary logistic regression model, with death as a dependent variable and using the following explanatory dichotomous variables: age (<68 years versus ≥68 years, the intensity of hospital department (low versus intermediate–high), days between symptoms onset and CCP transfusion (<7 versus ≥7) and CCP neutralizing titer (<320 versus ≥320). Calculations were performed with IBM SPSS Statistics software version 24.

## 3. Results

The baseline demographic and clinical characteristics of the 405 COVID-19 patients receiving CCP during the 12-month period of the study are reported in Table 1. All patients were of Caucasian ethnicity. The median age was 68 years (IQR, 56–78 years), with an excess of men over women (male/female ratio: 1.6). The patients’ median body mass index (BMI) at enrollment was above the normal range (25.7; IQR 23.4–31.0), and more than half of them (153/278, 55.0%) were overweight or obese. Approximately a quarter of patients (83/324, 25.6%) had three or more comorbidities, classified as follows in order of frequency: hypertension (56.8%), dyslipidemia (33.0%), cardiovascular disease (29.6%), diabetes (21.6%), chronic lung disease (11.7%), cancer (10.2%) and chronic kidney disease (9.6%). Regarding the degree of COVID-19 severity, 30.6% (124/405) of patients were admitted to intermediate/high-intensity departments, a proportion similar to that of the more severe forms of COVID-19 (PaO2/FiO2 ≤ 150: 34.8% (141/405)). Thus, the intensity of the hospital department appeared to be a reliable surrogate of a patient’s disease severity.

Almost all patients (388/405, 95.8%) were under heparin-based anticoagulation at the time of CCP infusion. A substantial proportion of them was also receiving corticosteroids (332/405, 82.0%) and antibiotics (264/405, 65.2%). Thirty-nine percent of patients (158/405) belonged to O blood type, a percentage (39%) that is slightly lower than that observed in the population of healthy blood donors from the same geographical area (43.6%) [14]. These results are in accordance with previous evidence of a protective effect of O blood type against COVID-19 [14].

Regarding the CCP-related data, the median time between symptom onset and CCP therapy was 7.5 days (IQR 5–12 days), and the mean number of CCP units infused per patient was 1.6 (±0.6) units. Overall, 657 CCP units, with a median nAb titer of 160 (IQR 80–320), were transfused to the 405 COVID-19 patients. Of them, 181 patients (44.7%) received 1 unit; 195 patients (48.1%), 2 units; 28 patients (6.9%), 3 units; and 1 patient (0.3%), 4 units. The median CCP neutralizing titer was 160 (IQR 80–320). Seventy-nine patients (19.5%) were treated during the first pandemic wave (driven by multiple D614G strains), while most of them (326/405, 80.5%) received CCP during the second/third pandemic wave (driven in Italy by the SARS-CoV-2 Alpha variant of concern).

**Figure 1:**
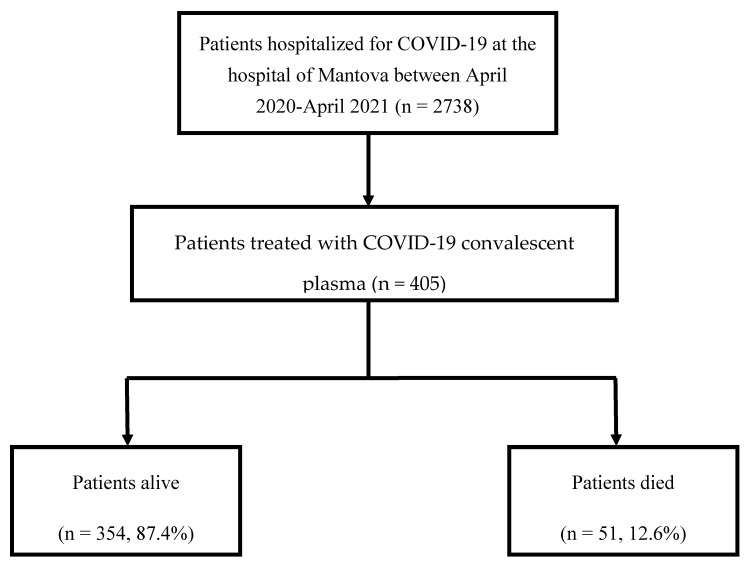
Flowchart of patients’ enrollment.

The 28-day crude mortality rate among the 405 CCP-treated patients was 12.6% (51/405) (Figure 1). A slightly higher number of deaths was recorded during the first pandemic wave as compared with the second-third ones (27/51 (52.9%) versus 24/51 (47.1%), P = NS). A statistically significant difference in the overall mortality rate was observed between CCP-treated patients and the entire cohort of patients admitted to the city hospital of Mantova for COVID-19 during the same period (51/405 (12.6%) versus 510/2738 (18.6%), P 0.003) [15].

Regarding the safety of CCP, 9 (1.3%) adverse reactions were recorded out of a total of 657 CCP units transfused. All cases were mild allergic reactions characterized by pruritus or rash, which rapidly faded with slowing of the CCP transfusion and after treatment with intravenous administration of antihistamine agents. In no case was it necessary to discontinue CCP transfusion. No cases of TRALI or other serious adverse events were recorded.

Table 2 reports a subgroup analysis that compared various variables in CCP-treated patients who survived (n = 354) or died (n = 51). Among deceased patients, 74.5% (38/51) were aged 68 years or older. At univariate analysis (died versus survived patients), death was significantly associated with an older median age (77 years versus 66 years, *p* < 0.001), a higher median BMI (31.1 Kg/m^2^ versus 24.7 Kg/m^2^, *p* < 0.001), a greater number of associated comorbidities (≥3 comorbidities: 74.2% versus 20.5%, *p* < 0.001), a more advanced disease (measured as median PaO2/FiO2 (92.0 versus 169.5, *p* < 0.001) and a higher intensity of hospital department (intermediate–high intensity: 49% versus 28%, *p* = 0.004)), in accordance with previous literature. In addition, deceased CCP-treated patients received CCP units later (16 days versus 7 days, *p* < 0.001) and with a less amount of nAb (mean nAb titer: 179.6 versus 227.2, *p* = 0.04) than alive patients. Interestingly, 82.3% (42/51) of deceased patients were transfused with CCP units with a nAb less than 320, while 90.2% (46/51) of them received CCP 7 days or more from the onset of symptoms. No deceased patient received CCP within 72 h from symptom onset. By contrast, no statistically significant difference between these two groups (alive and died) was observed regarding the sex and ABO blood group distribution and the mean number of CCP units transfused per patient.

The logistic regression model was statistically significant (Chi-squared test (5) = 43.102, *p* < 0.001). The model (Nagelkerke R^2^) explained 19.0% of the variance in mortality and correctly classified 87.4% of cases. Of the five predictor variables analyzed (age ≥ or < 68 years, sex, ≥ or < 7 days from symptom onset and CCP transfusion, low or intermediate/high intensity of hospital department, nAb titer ≥ or < 320), four were statistically significant: age, days between symptoms onset and CCP transfusion, intensity of hospital department and CCP nAb titer (as shown in Table 3). Multivariate analysis was not possible for the variables “BMI” and “≥ or <3 comorbidities” because of the lack of data recorded. Patients with age over 68 years had 3.45 times higher odds of dying. Increasing days between symptoms onset and CCP transfusion (≥7 days) were associated with an increased likelihood of dying. Similarly, higher intensity of hospital department was associated with mortality, but increasing CCP nAb titer (≥320) was associated with a reduction in the likelihood of dying.

## 4. Discussion

The first case of COVID-19 in Italy was diagnosed in the Lombardy region, in Codogno, on 20 February 2020. In the context of the region, there has subsequently been a diffusion of cases in a centrifugal direction starting from the Lodi area and from West to East. In this way, the first indigenous cases of the province of Mantua, at extreme East Lombardy, appeared a few days later (26 February). Thanks to this short delay, the health facilities of Mantua managed to develop an initial organizational adaptation to cope with the emergency by transforming the great majority of internal and surgery departments in COVID-19 areas [15]. In addition, to face the absolute lack at that time of effective treatments against this new serious infection, early efforts were directed towards the implementation of the collection of plasma from recovered individuals based on the first positive experiences from China and from previous pandemics [16,17,18]. We were the first center, along with the Hospital of Pavia, to use CCP in Italy and in western countries. Between the 12-month period (April 2020–April 2021) of the first three pandemic waves, more than 500 hundred CCP units were collected at the Transfusion center of Mantua hospital [19,20], allowing the treatment of 405 consecutive patients hospitalized for severe COVID-19 on the basis of a CCP compassionate use program.

The results of our cohort study are encouraging: the 12.6% mortality observed compares favorably with the overall mortality rate (18.6%) reported in COVID-19 patients hospitalized during the same period (April 2020–April 2021) in the same hospital [15]. Our data are in line with those recently published by the Registry of the Italian Veneto region [21], which confines with Lombardy region and Mantua province. During the one-year period (April 2020–April 2021), the investigators reported data analysis and clinical results in 1517 COVID-19 inpatients treated with high-titer CCP and observed a significantly reduced 30-day mortality in CCP-treated versus non-randomized, contemporary CCP-untreated patients (209/1517 (14%) versus 7700/31,071 (25%), *p* < 0.001). The mortality rate (13%) observed in the early experience on CCP EAP use in the USA [22], which led to the Emergency Use Authorization (EUA) by Food and Drug Administration (FDA), was very close to that reported in our study and the Veneto study.

Several factors, in our opinion, can be advocated to explain the discrepancy between mortality rates observed in real-life studies, as ours, and in RCTs, most of which failed to show a reduction in mortality and some discontinued for futility. Such reasons include the fact that CCP is not pharmaceutical but rather an artisanal product (it is produced at transfusion centers), and its nAb titer and absolute content in the cumulative volume varies widely [23]. Besides the CCP variability, differences in study design, patients’ characteristics and disease severity could have played a role. Nevertheless, we would like to highlight that most of the published RCT showed signals of CCP efficacy, including reductions in mortality, when subgroup analyses were restricted to the early use of high-titer CCP [8].

Notably, nearly 80% of patients in our cohort received CCP during the second–third wave, and this fact could have contributed, along with the general improvement of the management of COVID-19 patients (i.e., widespread use of steroids and remdesivir, optimization of oxygen therapy, etc.) to the reduction of mortality for COVID-19 observed during the different waves in our hospital in low-intensity departments (from 21.9% in the first wave to 12.7% in the third wave) in a recently published analysis [15]. A similar finding, i.e., a strong inverse correlation between CCP use and mortality per hospital admission, was also observed in a recent publication reporting the US experience on EAP use of CCP in approximately 500,000 patients [24].

Multivariate analysis in our study showed that younger patients (<68 years) with less severe COVID-19 (i.e., admitted in low-intensity departments), where those who benefited the most from hyperimmune plasma-based therapy, particularly when it was administered early (<7 days from symptom onset) and at very high titer (>320). The latter finding is quite interesting as it confirms the most recent evidence from the literature showing a significant benefit of CCP among COVID-19 patients who received a larger amount of nAb [8,25].

Another noteworthy issue of our study is the safety of CCP transfusion. Adverse reactions, all of which were mild, were observed only in 1.3% of CCP transfused. This rate is similar to that reported in our previous meta-analysis on CCP-related adverse reactions [26]. All concerns about CCP safety have, however, been swept away by the recent results of the US Expanded Access Program (EAP), which reported an incidence of serious adverse events of less than 1% in over 100,000 CCP-treated patients, definitely proving the overall high safety profile of this passive immunotherapy [27].

In our study, we found a reduced incidence of O blood type among COVID-19 patients as compared with the control healthy population. By contrast, ABO blood type was not found to be associated with a favorable outcome in CCP-treated patients. These results are in line with the published literature data showing that O blood type protects against SARS-CoV-2 infection (the receptor-binding domain of SARS-CoV-2 preferentially binds the blood group A expressed on respiratory epithelial cells) [28] but not against the degree of COVID-19 severity [29].

In conclusion, based on our experience, the use of CCP in hospitalized COVID-19 patients was characterized by a high safety and efficacy profile, particularly when administered early and at high nAb titer. In this regard, as recently reported [30,31], the ideal clinical setting for high-titer CCP use is probably the treatment of early outpatients to minimize the risk of hospitalization. In this regard, the US FDA has updated its emergency use authorization on 27 December 2021, expanding use to outpatients (available at: https://www.fda.gov/media/141477/download, accessed on 28 December 2021). Our experience, in conjunction with that of other centers, could help to plan a proper CCP emergency use in the case of future SARS-CoV-2 variants of concern or pandemics from different pathogens.

## Figures and Tables

**Table 1:** Demographic and clinical characteristics of the 405 patients enrolled in the study.

Parameters	Results
Median age, years (IQR)	68 (56–78)
Males/females	251/154
Male/female ratio	1.6
Median BMI (kg/m^2^) (IQR)	25.7 (23.4–31.0)
BMI (kg/m^2^), n (%) ^1^	
- Normal (18.5–24.9)	125 (45.0)
- Overweight (25.0–29.9)	71 (25.5)
- Grade 1 obesity (30.0–34.9)	51 (18.3)
- Grade 2 obesity (35–39.9)	23 (8.3)
- Grade 3 obesity (>40)	8 (2.9)
Associated comorbidities, n (%) ^2^	
- Hypertension	184 (56.8)
- Dyslipidemia	107 (33.0)
- Cardiovascular disease	96 (29.6)
- Diabetes	70 (21.6)
- Chronic lung disease	38 (11.7)
- Cancer	33 (10.2)
- Chronic kidney disease	31 (9.6)
- <3 comorbidities	241 (74.4)
- >3 comorbidities	83 (25.6)
COVID-19 severity, n (%)	
- PaO2/FiO2 ^3^	
> 200–300	76 (18.8)
100–200	281 (69.4)
< 100	48 (11.8)
- Hospital department	
Low intensity	281 (69.4)
Intermediate/high intensity	124 (30.6)
Concomitant therapies, n (%)	
- Antiviral agents ^4^	84 (20.7)
- Antibiotics	264 (65.2)
- Hydroxychloroquine	52 (12.8)
- Steroids	332 (82.0)
- Anticoagulants ^5^	388 (95.8)
Median interval between symptoms onset and CCP therapy, days (IQR)	7.5 (5–12)
ABO blood type, n (%)	
- O	158 (39.0)
- A	188 (46.4)
- B	44 (10.9)
- AB	15 (3.7)

Abbreviations: IQR, interquartile range; BMI, body mass index; CCP, COVID-19 convalescent plasma. ^1^ Data available on 278 patients. ^2^ Data available on 324 patients. ^3^ Measured before convalescent plasma transfusion. ^4^ Protease inhibitors and remdesivir. ^5^ Low molecular weight heparin.

**Table 2:** Subgroup analysis between CCP-treated patients alive and died.

Parameters	Alive n = 354 (87.4%)	Died n = 51 (12.6%)	*p*
Median age, years (IQR)	66 (55.2–76.3)	77 (67.8–81.8)	<0.001
Sex (males/females), number (ratio)	218/136 (1.6)	33/18 (1.8)	NS
Median BMI (kg/m^2^) (IQR)	24.7 (22.9–28.6)	31.1 (27.5–35–5)	<0.001
Comorbidities, n (%) - <3 - ≥3	233/293 (79.5) 60/293 (20.5)	8/31 (25.8) 23/31 (74.2)	<0.001
PaO2/FiO2, median (IQR)	169.5 (139.2–231.0)	92.0 (67.0–138.0)	< 0.001
PaO2/FiO2, n (%) ^1^ - <150 - ≥150	122/354 (34.5) 232/354 (65.5)	38/51 (74.5) 13/51 (25.5)	<0.001
Hospital department, n (%) - Low intensity - Intermediate/high intensity	255/354 (72.0) 99/354 (28.0)	26/51 (51.0) 25/51 (49.0)	0.004
ABO blood type, n (%) - O - Non-O	137/354 (38.7) 217/354 (61.3)	21/51 (41.2) 30/51 (58.8)	NS
Days between symptoms onset and CCP therapy, median (IQR)	7 (4–10)	16 (11–29.5)	<0.001
Days between symptoms onset and CCP therapy - <7 - ≥7	126/354 (35.6) 228/354 (64.4)	5/51 (9.8) 46/51 (90.2)	<0.001
Days between symptoms onset and CCP therapy - <3 - ≥3	39/354 (11.0) 315/354 (89.0)	0/51 (0) 51/51 (100)	0.01
CCP units transfused, mean (±SD)	1.6 (±0.6)	1.7 (±0.8)	NS
CCP neutralizing titer, mean (±SD)	227.2 (±184.7)	179.6 (±170.3)	0.04
CCP neutralizing titer <160 ≥160	116/354 (32.8) 238/354 (67.2)	23/51 (45.1) 28/51 (54.9)	NS
CCP neutralizing titer <320 ≥320	239/354 (67.5) 115/354 (32.5)	42/51 (82.3) 9/51 (17.7)	0.03

Abbreviations: NS, not significant; CCP, COVID-19 convalescent plasma; BMI, body mass index; SD, standard deviation. ^1^ Measured before convalescent plasma transfusion.

**Table 3:** Logistic regression predicting likelihood of death based on age, days between symptoms onset and CCP transfusion, intensity of hospital department and CCP neutralizing titer.

	B	SE	Wald	df	*p*	OR	95% CI	
Sex	−0.052	0.338	0.023	1	0.879	0.950	0.490	1.841
Age (≥68 years)	1.239	0.355	12.215	1	0.000	3.452	1.723	6.916
Days between symptoms onset and CCP transfusion (≥7 days)	1.589	0.496	10.260	1	0.001	4.897	1.853	12.945
Intensity of hospital department (high)	1.115	0.336	11.046	1	0.001	3.051	1.580	5.890
CCP neutralizing titer (≥320)	−0.829	0.412	4.052	1	0.044	0.437	0.195	.978
Constant	−4.141	0.590	49.246	1	0.000	0.016		

Abbreviations: OR, odds ratio; df, degrees of freedom; SE, standard error; CI, confidence interval.

## Data Availability

The data presented in this study are available on request from the corresponding author.

## Supplementary Materials

The following are available online at https://www.mdpi.com/article/10.3390/life12030420/s1.
