# Supplementary material for: Convalescent Plasma for Hospitalized COVID-19 Patients: A Single-Center Experience
Source: Life (Basel). 2022 Mar 14;12(3):420. doi: 10.3390/life12030420 (PMC8950373; doi:10.3390/life12030420)
Supplement: Supplementary file 1 [file life-12-00420-s001.zip › life-1626198-supplementary.pdf]

life

Supplementary Material of Convalescent Plasma for Hospitalized COVID-19 Patients: A Single-Center Experience

Convalescent Plasma Study Group

Consuelo Basili,[1] Angela Bellani,[2] Alberto Biondo,[3] Enrico Burato,[4] Irene Breoni,[5] Beatrice Caruso,[6] Maria Beatrice Casarini,[5] Maria Giovanna Cavazzini,[7] Margherita Chiamenti,[7] Nicola Cicorella,[8] Paolo Costa,[7] Giovanni Carlo Fibbia,[7] Elisa Firriolo,[9] Sabrina Frattima,[7] Rino Frizzelli,[5] Angela Furini,[10] Silvia Garilli,[7] Gianni Gattuso,[7] Susy Longhi,[11] Francesca Losa,[12] Claudio Martini,[5] Fabia Mascaro,[11] Linda Maulucci,[5] Barbara Melara,[10] Elena Miglioli,[13] Alessandro Occhionero,[7] Roberto Olivetti,[5] Matteo Ortolani,[3] Solidea Palin,[14] Loredana Palvarini,[7] Giorgio Perboni,[7] Viviana Ravagnani,[12] Francesca Saggiani,[5] Cinzia Sissa,[15] Piero Superbi,[16] Marcello Vincenzi,[7] Baoran Yang,[12] Fabio Zacchi,[7] Paolo Zampriolo.[14]

[1]Medical Direction, Policlinico S. Orsola-Malpighi, Bologna, Italy;

[2]Department of Frailty, Carlo Poma Hospital, Azienda Socio Sanitaria Territoriale of Mantova, Italy;

[3]Department of Anesthesiology and Intensive Care, Carlo Poma Hospital, Azienda Socio Sanitaria Territoriale of Mantova, Italy;

[4]Quality, Accreditation and Risk Management Unit, Azienda Socio Sanitaria Territoriale, Mantova, Italy;

[5]Department of Medicine, Carlo Poma Hospital, Azienda Socio Sanitaria Territoriale of Mantova, Italy;

[6]Laboratory, Carlo Poma Hospital, Azienda Socio Sanitaria Territoriale, Mantova, Italy;

[7]Unit of Infectious Diseases, Carlo Poma Hospital, Azienda Socio Sanitaria Territoriale of Mantova, Italy;

[8]Division of Cardiology, Carlo Poma Hospital, Azienda Socio Sanitaria Territoriale of Mantova, Italy;

[9]Management Planning and Control Service, Carlo Poma Hospital, Azienda Socio Sanitaria Territoriale of Mantova, Italy;

[10]Emergency Department, Carlo Poma Hospital, Azienda Socio Sanitaria Territoriale of Mantova, Italy;

[11]Intensive Cardiopulmonary Rehabilitation Unit, Carlo Poma Hospital, Azienda Socio Sanitaria Territoriale of Mantova, Italy;

[12]Allergology and Clinical Immunology Unit, Department of Medicine, Carlo Poma Hospital, Azienda Socio Sanitaria Territoriale of Mantova, Italy;

[13]Press and Communication Office, Azienda Socio Sanitaria Territoriale of Mantova, Italy;

[14]Department of Obstetrics and Gynecology, Carlo Poma Hospital, Azienda Socio Sanitaria Territoriale of Mantova, Italy;

[15]Department of Transfusion Medicine and Hematology, Carlo Poma Hospital, Azienda Socio Sanitaria Territoriale of Mantova, Italy;

[16]Medical Direction, Azienda Socio Sanitaria Territoriale of Mantova, Italy.
